# Readily Available Sources of Long-Chain Omega-3 Oils: Is Farmed Australian Seafood a Better Source of the Good Oil than Wild-Caught Seafood?

**DOI:** 10.3390/nu6031063

**Published:** 2014-03-11

**Authors:** Peter D. Nichols, Brett Glencross, James R. Petrie, Surinder P. Singh

**Affiliations:** 1 Commonwealth Scientific Industrial Research Organization, Food Future Flagship, Marine and Atmospheric Research, GPO Box 1538, Hobart, TAS 7000, Australia; 2 Commonwealth Scientific Industrial Research Organization, Food Future Flagship, Marine and Atmospheric Research, EcoSciences Precinct, 41 Boggo Road, Dutton Park, QLD 4102, Australia; E-Mail: brett.glencross@csiro.au; 3 Commonwealth Scientific Industrial Research Organization, Food Futures Flagship, Division of Plant Industry, P.O. Box 1600, Canberra, ACT 2601, Australia; E-Mails: james.petrie@csiro.au (J.R.P.); surinder.singh@csiro.au (S.P.S.)

**Keywords:** aquaculture, Atlantic salmon, barramundi, lipids, long-chain omega-3, EPA, DHA

## Abstract

Seafood consumption enhances intake of omega-3 long-chain (≥C_20_) polyunsaturated fatty acids (termed LC omega-3 oils). Humans biosynthesize only small amounts of LC-omega-3, so they are considered semi-essential nutrients in our diet. Concern has been raised that farmed fish now contain lower LC omega-3 content than wild-harvested seafood due to the use of oil blending in diets fed to farmed fish. However, we observed that two major Australian farmed finfish species, Atlantic salmon (*Salmo salar*) and barramundi (*Lates calcifer*), have higher oil and LC omega-3 content than the same or other species from the wild, and remain an excellent means to achieve substantial intake of LC omega-3 oils. Notwithstanding, LC omega-3 oil content has decreased in these two farmed species, due largely to replacing dietary fish oil with poultry oil. For Atlantic salmon, LC omega-3 content decreased ~30%–50% between 2002 and 2013, and the omega-3/omega-6 ratio also decreased (>5:1 to <1:1). Australian consumers increasingly seek their LC omega-3 from supplements, therefore a range of supplement products were compared. The development and future application of oilseeds containing LC omega-3 oils and their incorporation in aquafeeds would allow these health-benefitting oils to be maximized in farmed Australian seafood. Such advances can assist with preventative health care, fisheries management, aquaculture nutrition, an innovative feed/food industry and ultimately towards improved consumer health.

## 1. Introduction

The health benefits of omega-3 long-chain (≥C_20_) polyunsaturated fatty acids (LC-PUFA, also termed LC omega-3 oils) were first documented over three decades ago. Scientists observed that Greenland Eskimos had lower incidence of heart disease than other ethnic groups despite their high fat diet that was rich in the blubber of marine mammals [1]. The main LC omega-3 oils that have been attributed to this health benefit are eicosapentaenoic acid (EPA, 20:5ω3) and docosahexaenoic acid (DHA, 22:6ω3). For brevity, we use the term LC omega-3 here in consideration of both fatty acids, and also that of docosapentaenoic acid (DPA, 22:5ω3). Seafood has traditionally been the major source of these health-benefitting LC omega-3 oils [2]. Over the past decade the supply of farmed seafood has steadily increased, with the contribution of aquaculture to human food supplies now similar in volume to that of the wild catch harvest.

Aquaculture is currently the main user of industrially produced fish oils, with around 90% of global fish oil production used in aquafeeds [3]. Fish oils are used in feeds for most aquaculture species to satisfy both essential fatty acid and energetic demands [4]. However, as aquaculture has expanded, the ability of existing supplies, including the increasing cost, of the wild harvest fish oil resource to meet industry needs has been largely surpassed [5]. Non-marine sources of oil, including vegetable and animal-derived, are therefore now also included in aquafeeds as alternatives to fish oil. However, both alternatives to fish oil have a lower content of LC omega-3 oil, which has the flow on effect of causing a lower concentration of LC omega-3 in farmed seafood products compared to that observed previously [6]. Generally Australian consumers and also, to our knowledge, consumers in most other countries are not fully aware of the lower LC omega-3 content now occurring in many farmed fish species. In addition to the trend of fish oil replacement largely by oil blends, other potential strategies exist, including finishing diets used prior to harvest and novel ingredients such as LC omega-3 precursors [7]. However, to ensure the long-term supply of LC omega-3 oils for aquaculture there is a clear need for new and sustainable sources of these oils from fishery independent sources.

Obtaining information on the composition of farmed seafood products is important for future developments within the aquaculture and associated feed manufacturing industries. This study was initiated to examine the fatty acid content and composition of two major Australian farmed fish species—Atlantic salmon (*Salmo salar*) and barramundi (*Lates calcifer*). Particular emphasis was given to the LC omega-3 oils, and also the comparison of the changes seen in the fatty acid profiles between samples collected over 2010–2013 and earlier data for these same species obtained during 2002 when a largely fish oil diet was in use. As many Australian consumers increasingly seek their LC omega-3 from supplements, a range of supplement products also were examined and compared.

## 2. Experimental Section

### 2.1. Sample Collection

Norwegian quality cut (NQC) samples from three fresh fillets of farmed Atlantic salmon were obtained twice per year between December 2010 and November 2013. At CSIRO, the skin was removed and each sample was independently blended for two minutes in an OSKAR 400 continuous flow homogenizer. Whole barramundi were supplied to CSIRO in November 2010 by Marine Produce Australia Pty Ltd. (Broome, WA, Australia). The right fillet of each of three fish was used and the NQC analogous cut from that fillet of the barramundi was taken. The NQC sample was then minced, with a sample analysed for moisture content by gravimetric analysis after oven drying at 105 °C for 24 h. The remainder of the sample was frozen, freeze dried, ground in a coffee grinder, and then shipped to Hobart for lipid extraction and analysis.

Fourteen commercially produced omega-3 fish oil capsules were purchased from a local Hobart pharmacy. Each product was given a laboratory code: FO1-14. Oil capsules were cut open with a scalpel and the oil transferred to a glass vial using a glass pipette.

### 2.2. Lipid Extraction

NQC samples (typically between 1 and 3 g wet weight for Atlantic salmon, dry weight for barramundi) were quantitatively extracted overnight using a modified Bligh and Dyer [8] single-phase methanol-chloroform-water extraction (2:1:0.8 v/v/v). The phases were separated by addition of chloroform-water (final solvent ratio, 1:1:0.9 v/v/v methanol-chloroform-water). The total solvent extract (TSE) was concentrated using rotary evaporation at 40 °C, and total lipid content was determined gravimetrically.

### 2.3. Fatty Acid Analysis

An aliquot of the TSE of the farmed fish samples or the fish oils dissolved in chloroform was trans-methylated to produce FA methyl esters (FAME) using methanol–chloroform–conc. hydrochloric acid (3 mL, 10:1:1, 80 °C, 2 h) [9]. FAME were extracted into hexane–chloroform (4:1, 1.8 mL). The samples were dried on a heat block (40 °C) under a stream of nitrogen gas, and an internal injection standard (C_19_ or C_23_ FAME) added.

Samples were analysed by gas chromatography (GC) using an Agilent Technologies 7890A GC (Palo Alto, CA, USA) fitted with a Supelco Equity™-1 fused silica capillary column (15 m × 0.1 mm ID, 0.1 μm film thickness (Bellefont, PA, USA) an FID, a split/splitless injector and an Agilent Technologies 7683B Series auto sampler and injector. Helium was the carrier gas. Samples were injected in splitless mode at an oven temperature of 120 °C. After injection, the oven temperature was raised to 250 °C at 10 °C min^−1^ and finally to 270 °C at 3 °C min^−1^. Peaks were quantified with Agilent Technologies ChemStation software (Palo Alto, CA, USA). GC-mass spectrometric (GC-MS) analyses of selected samples were performed on a Finnigan Thermoquest GCQ GC-MS fitted with an on-column injector using Thermoquest Xcalibur software (Austin, TX, USA). The GC was fitted with a capillary column of similar polarity to that described above. Individual components were identified using mass spectral data and by comparing retention time data with those obtained for authentic and laboratory standards.

## 3. Results

### 3.1. Oil Content

Lipid content of the farmed barramundi samples averaged 10% (WW) in 2002 and 8.5% in 2010 (wet weight, WW). Oil content of the farmed Atlantic salmon samples ranged from 6.7% to 14.7% (WW). The 2010 and 2011 autumn Atlantic salmon samples contained lower oil content than all other samples collected from 2010 to 2013.

### 3.2. Fatty Acid Composition and Content

#### 3.2.1. Farmed Fish

Major fatty acids (FA) (as % of the total fatty acids, TFA) in farmed Australian Atlantic salmon harvested in 2002 were in decreasing order of abundance: 16:0 (18%), DHA (17%), 18:1ω9 (oleic acid, OLA; 15%), EPA (10%) and 16:1ω7 (6%) (Table 1). The three LC omega-3 PUFA—EPA + DPA + DHA accounted for 30% of the TFA.

For farmed Atlantic salmon obtained in 2010–2013, the FA profile differed both from the 2002 sample, and also over the 4 year period. Major FA in the 2010–2013 samples in decreasing order of abundance were: OLA, 16:0, 18:2ω6 (linoleic acid, LOA) and 16:1ω7 (Table 1); these four FA accounted for 57% (autumn 2010) rising to 72% of TFA in late spring 2013. In 2002, these four FA had accounted for 42% of TFA in farmed salmon. The next most abundant FA in the 2010–2013 Atlantic salmon samples were: EPA, DHA and 18:0, with all three FA decreasing over this period. LOA increased from around 2.5-fold (autumn 2010) to 4-fold (spring 2013) compared to the 2002 sampling, and the relative proportion of arachidonic acid (ARA) decreased over the 2010–2013 period.

**Figure 1:**
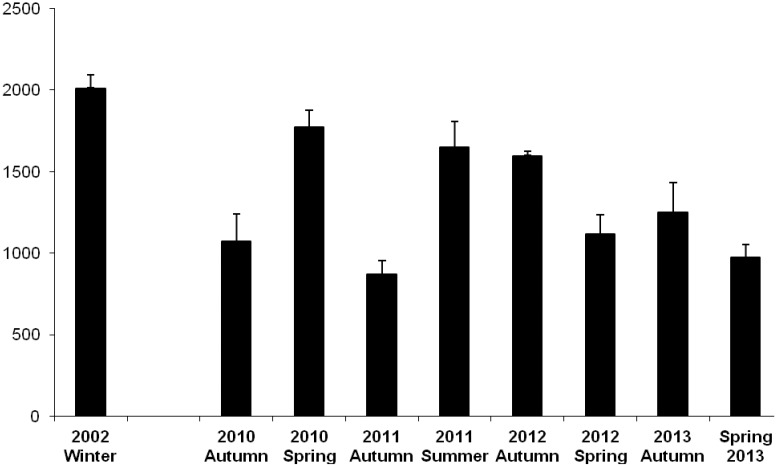
Content (±SD) of LC omega-3 oils (EPA + DPA + DHA, mg/100 g) in farmed Tasmanian Atlantic salmon sampled in 2002 [10] and 2010–2013.

**Figure 2:**
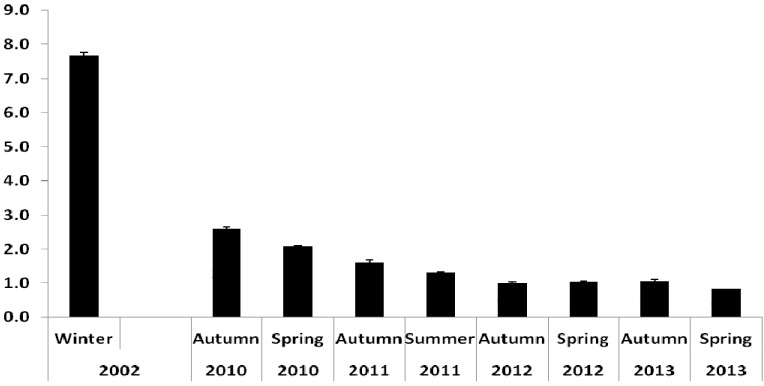
Ratio (±SD) of omega-3 PUFA/omega-6 PUFA in farmed Tasmanian Atlantic salmon, 2002 [10] and 2010–2013.

Expressed on an absolute basis (mg/100 g serve, WW), LC omega-3 content in farmed Atlantic salmon was 2010 mg/100 g in 2002, then ranged from as high as 1770 mg/100 g (spring 2010) decreasing to 980 mg/100 g in spring 2013 (Figure 1). The ratio of omega-3 PUFA/omega-6 PUFA was 7.8 in 2002 samples, and through 2010 to 2013 showed a steady decrease from 2.6 (autumn 2010) to 0.8 (spring 2013) (Figure 2).

The FA profiles of farmed and wild caught barramundi are shown in Table 2. Major FA (as % TFA) in farmed samples from 2010 in decreasing order of abundance were: OLA, 16:0, LOA and 16:1ω7; these four FA accounted for approximately 60% of TFA in farmed barramundi. The next most abundant FA in farmed barramundi in 2010 were: EPA, DHA and 18:0. The relative level values for DHA in particular are lower than recorded for the 2002 farmed samples.

**Table 1:** Composition of fatty acids (as percent of total FA) in farmed Atlantic salmon (*n* = 3)—2002 and 2010–2013.

Fatty acid	Sample Date								
	2002	2010	2010	2011	2011	2012	2012	2013	2013
	Winter	Autumn	Spring	Autumn	Summer	Autumn	Spring	Autumn	Spring
14:0	5.5	2.1	2.7	2.8	2.5	2.4	1.8	1.9	1.7
15:0	0.5	0.2	0.2	0.3	0.2	0.2	0.2	0.2	0.2
16:4	0.3	0.7	0.6	0.5	0.5	0.3	0.2	0.3	0.2
16:3	0.4	0.8	0.6	0.6	0.5	0.4	0.2	0.4	0.3
16:1ω7c	6.2	8.3	7.0	7.4	7.2	6.4	6.8	6.9	4.9
16:0	18.0	14.4	15.1	13.9	15.7	15.4	15.5	14.7	13.0
17:1ω8c + a17:0	0.4	0.3	0.3	0.4	0.3	0.3	0.3	0.3	0.3
17:0	0.5	0.2	0.3	0.2	0.2	0.2	0.2	0.2	0.2
18:3ω6	0.1	0.1	0.2	0.2	0.2	0.2	0.2	0.2	0.2
18:4ω3	3.0	1.4	1.3	1.0	1.0	0.9	0.7	0.8	0.7
18:2ω6	2.8	7.2	8.2	8.4	10.0	12.8	9.6	9.8	12.4
18:3ω3	0.0	0.9	1.1	1.1	1.0	0.7	1.1	0.9	2.1
18:1ω9c	14.5	27.0	29.2	33.8	33.8	33.0	39.0	38.8	42.7
18:1ω7c	3.3	4.0	3.9	4.0	3.5	3.1	3.5	3.4	3.4
18:1ω5c	0.1	0.1	0.2	0.2	0.2	0.2	0.2	0.2	0.1
18:0	4.2	3.9	4.4	4.2	4.1	4.5	4.5	4.5	4.0
20:4ω6	0.8	0.8	0.7	0.6	0.5	0.3	0.4	0.5	0.4
20:5ω3	9.6	8.5	7.4	5.9	4.9	4.4	3.1	3.4	2.7
20:3ω6	0.2	0.4	0.3	0.3	0.4	0.4	0.4	0.4	0.4
20:4ω3	1.5	0.9	0.8	0.7	0.6	0.5	0.4	0.5	0.5
C20PUFA	0.0	0.0	0.2	0.2	0.2	0.2	0.2	0.2	0.0
20:2ω6	0.3	0.4	0.5	0.5	0.5	0.7	0.6	0.5	0.0
20:1ω9c	1.7	1.4	1.4	1.7	1.5	1.8	1.8	1.7	1.7
20:1ω7c	0.2	0.3	0.3	0.3	0.2	0.2	0.2	0.2	0.2
20:0	0.2	0.2	0.2	0.2	0.1	0.2	0.2	0.1	0.2
21:5ω3	0.6	0.4	0.4	0.3	0.3	0.3	0.2	0.2	0.1
22:5ω6	0.3	0.1	0.1	0.2	0.1	0.1	0.1	0.1	0.1
22:6ω3	17.0	7.8	6.7	5.0	5.1	5.4	4.8	4.9	4.0
22:4ω6	0.1	0.2	0.1	0.2	0.1	0.1	0.1	0.1	0.1
22:5ω3	3.8	3.8	2.8	2.5	2.2	1.9	1.4	1.6	1.2
22:1ω11c	0.3	0.3	0.3	0.3	0.3	0.6	0.2	0.1	0.1
22:1ω9c	0.2	0.1	0.1	0.2	0.1	0.2	0.2	0.2	0.2
24:5ω3	0.0	0.2	0.2	0.0	0.2	0.1	0.1	0.1	0.0
24:1ω11c	0.0	0.1	0.0	0.1	0.0	0.0	0.0	0.0	0.0
24:1ω9c	0.5	0.2	0.2	0.0	0.2	0.2	0.2	0.1	0.2
24:0	0.0	0.0	0.0	0.2	0.0	0.0	0.0	0.0	0.0
Other	2.7	2.0	1.8	1.5	1.4	1.4	1.4	1.5	1.4
Sum	100.0	100.0	100.0	100.0	100.0	100.0	100.0	100.0	100.0
Sum SFA	29.4	21.9	23.4	22.4	23.2	23.3	22.7	21.9	19.7
Sum MUFA	29.2	43.1	44.1	49.6	48.2	46.8	53.3	52.8	54.7
Sum Omega-3 PUFA	35.5	24.0	20.9	16.5	15.4	14.3	11.9	12.4	11.3
Sum Omega-6 PUFA	4.6	9.3	10.0	10.4	11.8	14.6	11.6	11.8	13.6

SFA, Saturated fatty acids; MUFA, monounsaturated fatty acids; PUFA, polyunsaturated fatty acids. Prefix a denotes anteiso branching.

Major fatty acids in wild caught barramundi were 16:0, OLA, 18:0, DHA and ARA (Table 2). Less abundant components included LOA, 16:1ω7c, EPA, 18:1ω7c and DPA. Freshwater samples had higher relative levels of LOA, OLA, 16:1ω7c, 18:1ω7c, 18:3ω3 (α-linolenic acid, ALA), 14:0 and lower EPA and DHA than the saltwater specimens. The greatest difference between the wild fresh and saltwater samples for any single FA was observed for DHA (5%, freshwater; 22%, saltwater). Saltwater fish contained higher levels of LC omega-3 oils than freshwater fish. In addition to containing considerably higher relative levels of DHA, the saltwater fish contained higher relative levels of total PUFA than freshwater fish (Table 2). The relative level of ω6 PUFA was similar in freshwater barramundi and saltwater fish. In freshwater fish ARA made up 7.1% of TFA, while in the saltwater wild fish it made up 12.2% of TFA. In contrast, in farmed barramundi collected in 2002 and 2010, ARA levels were consistent at 0.6% to 0.7% and an order of magnitude lower. The ratio of ω3 PUFA/ω6 PUFA differs between salt (1.9) and freshwater (0.8) barramundi (Table 2).

**Table 2:** Composition of fatty acids (as percent of total FA) in wild and farmed barramundi.

Fatty Acid	1998	1998	2002	2010
	Freshwater	Saltwater	Farmed	Farmed
14:0	2.8	0.9	5.9	3.6
15:0	0.9	0.6	0.6	0.3
16:1ω7c	7.4	2.1	6.5	7.5
16:0	27.8	22.4	19	17.7
17:1ω8c + a17:0	0.9	0.7	0.5	0.3
18:3ω6	0.4	0.3	0.3	0.3
18:4ω3	0.4	0.6	1.9	1.3
18:2ω6	4.8	1	5.5	9.8
18:3ω3	1	0.2	0	1.1
18:1ω9c	17.9	11.7	19.6	26.2
18:1ω7c	4	2.1	2.9	3.2
18:0	8.8	12.5	4.4	4.8
20:4ω6	7.1	12.2	0.6	0.7
20:5ω3	1.3	3.1	6.2	6.9
20:3ω6	0.5	0.2	0.1	0.2
20:4ω3	0.3	0	0.8	0.5
20:2ω6	0.3	0.1	0.2	0.2
20:1ω9c	0.6	0.3	3.5	1.1
20:1ω7c	0.1	0	0.3	0.2
20:0	0.3	0.2	0.2	0.3
22:5ω6	0.4	0	0.3	0.2
22:6ω3	6.1	21.6	10.2	5.4
22:4ω6	1.2	1.2	0.1	0.1
22:5ω3	1.6	2.2	2.5	2.1
22:1ω11c	0	0	1.6	0.5
22:1ω9c	0	0	0.5	0.1
24:1ω9c	0.2	0.8	0.5	0.2
Other	2.9	3	5.3	2.3
Sum SFA	42.7	39.3	31.1	27.9
Sum MUFA	31.7	17.7	37	40.7
Sum Omega-3 PUFA	11.1	28.1	21.6	17.9
Sum Omega-6 PUFA	14.5	14.9	7.2	10
Omega-3/Omega-6	0.8	1.9	3	1.55

2002 farmed barramundi [10]. 1998 and 2002 samples analysed using identical methods to 2010 fish. Wild caught barramundi data [11]. Abbreviations as used in Table 1. Prefix a denotes anteiso branching.

**Figure 3:**
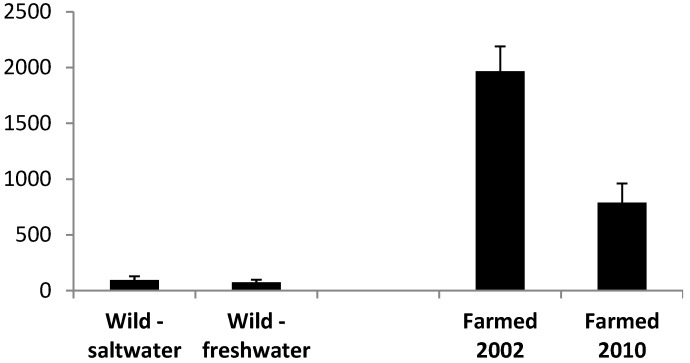
Content (±SD) (mg/100 g) of LC omega-3 oils (EPA + DPA + DHA) in wild caught (1998) [11] and farmed barramundi (2002 [10], 2010).

Absolute concentration data (mg/100 g wet weight) for the LC omega-3 oils in wild harvest barramundi are shown in Figure 3. Wild saltwater fish contained higher DHA levels. For farmed barramundi, LC omega-3 content (EPA + DPA + DHA) was markedly higher than in the wild harvest samples, in 2002, although the content decreased to 790 mg/100 g in 2010 (Figure 3).

#### 3.2.2. Fish Oil Capsules

The capsule samples were divided into three groups of LC omega-3 containing products. The first group was those products containing elevated EPA + DHA (FO1-5, Table 3); these products contained ≥500 mg of EPA + DHA per capsule, which represents use of enrichment processes to obtain the product. One product reached this grouping largely based on its larger capsule size (Cenovis Fish Oil Plus, FO4, 1500 mg) compared with all other products. The second group of capsules (FO6-9) was the brands containing 180 mg EPA and 120 mg DHA per 1000 mg capsule. The Cenovis Fish Oil Plus (FO4) 1500 mg capsule product, when normalized to 1000 mg, contained the same DHA content as the other group 2 oils purchased, with EPA higher than for the other group 2 products. The third group (FO10-14) contained lower EPA + DHA content than group 1 and 2 oils, ranging from 52 to 160 mg per capsule.

The FA profiles of the fish oil capsule products are shown in Table 4. All fish oil capsule supplements examined generally contained EPA + DHA at levels indicated on the product labels. The five group 1 fish oils contained 41%–78% EPA+DHA. The four group 2 oils—containing 180 mg EPA + 120 mg DHA—were very similar in composition containing around 30% EPA + DHA. The group 3 oils contained 16%–25% EPA + DHA.

Other major FA present in group 2 and 3 oils included the saturated FA (SFA)—16:0, 18:0 and 20:0, the monounsaturated FA (MUFA)—OLA, 18:1ω7c and 16:1ω7c. Several group 3 oils—wild salmon oil and cod-liver oil—both contained elevated levels (~18%) of LC-MUFA—20:1ω11c (salmon oil), 20:1ω9 (cod liver oil) and 22:1ω11c (Table 4)—distinguishing these two oils readily from other group 2 and 3 oils. The group 1 PUFA-enriched oils varied in composition. The Omega Brain oil (FO2) was elevated in DHA, with the other four group 1 products (FO1, FO3, FO4, FO5) each containing EPA > DHA. Products FO3 (Healthy Care Fish Oil One a Day) and FO5 (Natures Own Omega-3 Ultra) showed similar profiles, with 32%–36% EPA and 23% DHA. The omega-3 joint product (FO1) contained the highest EPA+DHA levels (78%), with a number of other PUFA present (18%); this oil contained the highest EPA/DHA ratio (3.4), and represents a highly purified PUFA-containing oil product compared with all other products.

**Table 3:** Fish oil capsules—brand and composition details as supplied by manufacturers.

Brand	Description	Lab Code	Capsule Size (mg)	Number of Capsules	Cost ($)	Cost ($) per Capsule	EPA mg per Capsule	DHA mg per Capsule	EPA + DHA mg per Capsule	Number of Caps for 500 mg EPA + DHA
**Group 1**										
Blackmores	Omega Joint	FO1	1000	60	22.39	0.37	550	120	670	1
Blackmores	Omega Brain	FO2	1000	60	22.39	0.37	100	500	600	1
Healthy Care	Fish oil One a Day	FO3	1000	50	14.99	0.30	360	240	600	1
Cenovis	Fish oil Plus	FO4	1500	62	16.69	0.27	335	185	520	2
Natures Own	Omega-3 Ultra	FO5	1000	60	22.69	0.38	302	201	503	1
**Group 2**										
Cenovis	Fish oil with Omega-3	FO6	1000	180	13.69	0.08	180	120	300	2
Blackmores	Odorless Fish Oil + Vitamin D3	FO7	1000	100	12.99	0.13	180	120	300	2
Healthy Care	Fish oil	FO8	1000	400	12.99	0.03	180	120	300	2
Swisse	Odorless Fish Oil	FO9	1000	200	22.99	0.11	180	120	300	2
**Group 3**										
Swisse	Wild salmon oil	FO10	1000	200	22.99	0.11	80	80	160	4
Blackmores	Pregnancy	FO11	1000	120	33.99	0.28	25	125	150	4
Natures Own	Fish oil + Glucosamine	FO12	1100	90	20.99	0.23	90	60	150	4
Swisse	Wild krill oil (NKO)	FO13	333	50	43.99	0.88	50	30	80	7
Cenovis	Cod liver oil *	FO14	275	90	3.99	0.04	23	29	52	10

* EPA + DHA data not supplied on label. Fatty acid data from [11].

**Table 4:** Fatty acid composition (as % of total fatty acids) of fish oil capsule products.

Sample	Group 1					Group 2				Group 3				
	FO1	FO2	FO3	FO4	FO5	FO6	FO7	FO8	FO9	FO10	FO11	FO12	FO13	FO14
14:0	0.1	0.2	0.9	4.2	0.5	5.7	5.8	5.7	6.2	4.8	6.9	6.6	9.1	3.3
15:0	0.0	0.1	0.0	0.3	0.0	0.5	0.4	0.5	0.5	0.4	0.7	0.4	0.4	0.3
16:4	2.7	0.1	0.4	2.2	0.4	2.2	2.4	2.3	2.2	0.5	0.1	1.8	1.3	0.4
16:3	1.8	0.1	0.4	1.6	0.2	1.5	1.8	1.5	1.6	0.5	0.1	1.4	0.3	0.3
16:1ω7c	0.5	0.8	1.7	6.9	1.4	9.6	9.4	9.6	9.7	6.8	3.8	8.0	6.8	6.6
16:1ω5c	0.0	0.0	0.0	0.1	0.0	0.2	0.2	0.2	0.2	0.4	0.1	0.2	0.5	0.3
16:0	0.3	3.2	2.6	11.3	3.5	15.1	15.9	15.0	15.6	13.4	18.5	15.5	19.7	10.9
i17:0	0.0	0.1	0.0	0.1	0.0	0.2	0.5	0.2	0.2	0.2	0.2	0.2	0.3	0.2
17:1ω8c + a17:0	0.0	0.2	0.1	0.2	0.1	0.4	0.4	0.4	0.4	0.5	0.6	0.3	0.4	0.7
17:0	0.0	0.3	0.1	0.2	0.2	0.5	0.4	0.4	0.4	0.3	0.8	0.4	0.1	0.2
18:3ω6	0.6	0.1	0.1	0.2	0.1	0.3	0.2	0.3	0.2	0.0	0.1	0.2	0.2	0.1
18:4ω3	8.1	0.7	1.5	2.2	2.0	3.3	3.3	3.3	3.3	2.4	0.8	2.7	3.9	2.3
18:2ω6	0.2	1.1	0.9	2.5	1.6	1.4	1.1	1.6	1.4	2.5	8.3	7.8	2.1	2.2
18:3ω3	0.2	0.4	0.6	0.6	0.6	0.8	0.6	0.8	0.8	0.9	1.2	1.4	1.0	0.7
18:1ω9c	0.2	7.7	6.6	7.8	10.5	8.5	10.0	8.4	8.4	16.8	12.7	10.0	12.6	20.0
18:1ω7c	0.0	1.7	2.8	3.2	3.0	3.9	3.5	3.9	4.0	4.0	2.3	3.7	8.4	4.7
18:1ω5c	0.0	0.1	0.1	0.1	0.1	0.1	0.1	0.2	0.1	0.6	0.1	0.2	0.4	0.4
18:0	0.1	3.9	2.8	2.7	3.5	3.4	3.2	3.4	3.5	2.4	7.8	5.3	1.4	2.6
20:4ω6	1.2	2.6	0.8	1.0	0.8	0.8	0.7	0.6	0.6	0.4	1.5	0.7	0.2	0.3
20:5ω3 EPA	60.4	14.5	35.6	27.4	31.8	18.9	20.0	19.0	18.7	9.1	5.4	15.1	16.9	8.8
20:3ω6	0.2	0.3	0.4	0.3	0.4	0.3	0.3	0.3	0.2	0.1	0.1	0.2	0.0	0.1
20:4ω3	0.9	0.9	1.8	1.2	1.5	1.0	0.9	1.0	1.0	1.2	0.4	0.8	0.4	0.8
C20PUFA	0.1	0.1	0.8	0.4	0.4	0.3	0.3	0.3	0.3	0.1	0.0	0.3	0.0	0.1
20:2ω6	0.1	0.6	0.5	0.3	0.4	0.3	0.2	0.4	0.3	0.3	0.2	0.3	0.0	0.3
20:1w11c	0.0	0.6	0.4	0.2	0.3	0.2	0.1	0.2	0.2	7.2	0.4	0.0	0.0	1.2
20:1ω9c	0.5	2.3	2.2	1.3	2.2	1.3	0.5	1.3	1.1	3.0	1.0	1.3	0.9	8.8
20:1ω7c	0.0	0.4	1.1	0.7	0.9	0.4	0.3	0.5	0.5	0.5	0.1	0.4	0.4	0.5
20:0	0.0	0.7	0.6	0.3	0.5	0.3	0.2	0.3	0.3	0.3	0.4	0.4	0.1	0.1
21:5ω3	1.5	0.6	1.4	1.1	1.6	0.7	0.8	0.7	0.8	0.4	0.2	0.6	0.5	0.4
22:5ω6	0.0	1.1	0.5	0.4	0.4	0.3	0.3	0.3	0.3	0.1	1.3	0.3	0.0	0.1
22:6ω3 DHA	17.7	46.8	23.2	13.2	23.2	11.8	10.9	11.6	11.7	6.9	18.8	9.4	8.2	10.3
22:4ω6	0.1	0.5	0.3	0.2	0.2	0.2	0.1	0.2	0.2	0.0	0.3	0.1	0.0	0.1
22:5ω3	1.5	2.9	4.5	2.9	4.3	2.3	2.1	2.3	2.2	1.8	1.2	1.8	0.4	1.2
22:1ω11c	0.0	0.6	1.6	0.9	1.0	0.7	0.1	0.7	0.6	7.6	0.5	0.5	0.0	7.5
22:1ω9c	0.0	0.4	0.4	0.2	0.3	0.2	0.1	0.1	0.2	0.7	0.1	0.1	0.5	0.4
22:0	0.0	0.4	0.2	0.2	0.2	0.1	0.3	0.1	0.1	0.1	0.2	0.2	0.0	0.1
24:6ω3	0.0	0.2	0.1	0.1	0.1	0.1	0.0	0.1	0.1	0.1	0.1	0.1	0.1	0.1
24:5ω3	0.0	0.1	0.1	0.1	0.1	0.1	0.0	0.1	0.1	0.1	0.0	0.1	0.1	0.2
24:1ω9c	0.0	1.0	0.7	0.4	0.6	0.4	0.3	0.4	0.4	0.6	0.7	0.4	0.4	0.3
24:0	0.0	0.3	0.1	0.0	0.1	0.1	0.1	0.1	0.1	0.0	0.4	0.1	0.0	0.0
Sum other	1.0	1.2	0.9	1.0	1.0	1.8	2.3	1.7	1.7	2.0	1.5	0.9	1.6	2.2
Sum	100	100	100	100	100	100	100	100	100	100	100	100	100	100
Sum EPA + DPA + DHA	79.7	64.3	63.2	43.5	59.4	33.0	33.0	32.9	32.6	17.8	25.4	26.3	25.5	20.2
Omega-3/Omega-6	16.6	9.3	11.2	4.9	9.9	6.8	7.4	6.7	7.4	4.5	2.0	1.9	6.2	5.8

Sample codes—refer to Table 3. Abbreviations as used in Table 1. Other FA (≤0.3% of total FA): 14:1ω5c, 4,8,12TMTD, i15:0, a15:0, i16:0, MBrFA:1, MBrFA, 17:1, C18PUFA, i18:0, 18:1ω7t, 18:1, 19:1, 20:1ω5c, 22:1ω7, 24:1ω11c.

## 4. Discussion

### 4.1. Farmed Fish—Oil Content and Fatty Acid Profiles

Total oil (lipid) content of farmed Atlantic salmon from 2010 to 2013 was generally similar to the oil content previously observed for Tasmanian Atlantic salmon [10]. Total lipid content in the whole body and fillet of most fish species, and barramundi are included in this regard, tends to increase with fish size [12]. In comparison, wild caught barramundi are considerably lower in lipid content (0.4% to 0.9%, WW; shoulder portion) [11]. Farmed barramundi fed a fish oil-based diet and previously analysed contained various concentrations of lipid according to study, diet and fish size and fillet section analyzed; around 10% (WW) lipid [10], 1.3% to 30% lipid [13] and 8% to 14% lipid [14].

The relative level (as % TFA) values for EPA and in particular DHA for the two farmed fish species sampled in 2010–2013 are lower than those recorded in the 2002 analyses when both species were being fed a fish oil based-diet (barramundi—EPA 6.2%, DHA 10.2%; Tasmanian Atlantic salmon—EPA 10%, DHA 17%) [10]. These 2010–2013 % composition values for EPA and DHA are also generally lower than those observed for most white muscle wild-caught fish species [11].

In addition to the reduction in EPA and DHA, the relative level of omega-6 PUFA was elevated in both farmed species sampled over the 2010–2013 period relative to 2002, due mainly to increased LOA. This leads to a dramatic shift in the ratio omega-3/omega-6 (*i.e.*, from >7:1 in 2002, to ≤1:1 currently for Atlantic salmon). A recent study [15] followed the release of an American Heart Association advisory on omega-6 fatty acids and cardiovascular risk [16] and has indicated—“advice to specifically increase omega-6 intake is unlikely to provide the intended benefits, and may actually increase the risk of CHD and death”. A further change in the FA profile of the two farmed fish is a large increase in the relative level of OLA. Collectively, this profile shift is reflecting a change in diet formulation to include higher proportions of terrestrial animal and plant-based oils, with concomitant reduction in fish oil. Similar changes in salmon oil quality occur in overseas markets [17]. Also notable over the 2002–2013 period was the overall decrease in SFA which are regarded as the “least healthy” and are known precursors for endogenous cholesterol synthesis. Therefore at least one element of the current fish oil replacement strategy could be argued as improving the nutritional quality of farmed fish. It is the absolute content of the LC omega-3 that is the most critical issue in terms of the contribution that farmed fish make to the human diet. On an absolute (mg/100 g serve) basis, the LC omega-3 content values observed for farmed Atlantic salmon and barramundi sampled in 2010–2013 are considerably higher than those found in most wild caught fish species [10,11], although the values from both species are lower than previously reported a decade ago in 2002 for these two farmed species fed a fish oil-based diet [10]. Large differences were observed between the autumn and spring/summer samples in 2010 and 2011. Tasmanian waters varied in water temperature through this period, together with the presence and degree of disease and other biological factors; these may be contributing factors to the observed differences.

Wild harvest barramundi contained considerably lower absolute amounts of PUFA than the famed samples (Figure 3). However, a low oil content combined with similar high relative levels of omega-3 in saltwater fish and both ω3 and ω6 PUFA in freshwater fish are nutritionally attractive features of the wild-harvest barramundi. The overall FA profile and lower LC omega-3 content of the wild specimens provide no major nutritional advantage in terms of these key components. Both wild specimens had an omega-3/omega-6 ratio similar to or poorer that that found in farmed barramundi.

Until about 10 years ago Australian and New Zealand fish farms were largely using feeds made with high inclusion of fishery products (fish meals and fish oils). Prior to this period, there was evidence that LC omega-3 content in farmed fish products remained high [2,3,10]. Increasing demand for fishery resources, such as fish oil, at a time where there is limited ability or capacity to increase sustainable harvest of wild fish stocks, has resulted in increased use of oils derived from non-traditional sources [7]. Oils now being routinely used include those from land plants (e.g., canola and palm oil) and rendered oils (e.g., poultry oil) [6]. The problem with the use of these oils is that they result in flesh with: (i) lower relative levels of LC omega-3 (as % of TFA); (ii) lower absolute content of the LC omega-3; and (iii) lower omega-3/omega-6 ratio. As the relative levels of these key LC omega-3 oils decrease, LOA and OLA increase. OLA and LOA are derived from the non-marine ingredients that are being increasingly substituted into aquafeeds, although most fish also have significant capacity to synthesis their own OLA from both lipid and non-lipid substrates [4].

Interest has existed in Australia and New Zealand on comparison of the LC omega-3 oil content of salmon farmed in the two locations. In Tasmania, the species farmed is Atlantic salmon (*Salmo salar*), whilst in New Zealand the predominant species is Chinook salmon (*Oncorrhynchus tshawytscha*, also termed king salmon). LC omega-3 content of the two species sampled at a similar time (spring 2012) showed Atlantic salmon containing 1117 ± 117 mg/100 g LC omega-3 with Chinook salmon at 2568 ± 153 mg/100 g. The two species generally receive similar diets [18], and the differences in LC omega-3 content (and similarly for the SFA and omega-6 PUFA) result largely from the higher oil content of the fillet of farmed Chinook salmon (~25% oil content cf 10%–15% in Atlantic salmon).

Atlantic salmon and barramundi have, when fed a FO-containing diet, provided an excellent source of beneficial omega-3 LC-PUFA for human consumption, but reduced concentrations of these nutrients, as occurs through the use of vegetable oil and/or animal fat diets, may reduce their nutritional benefit to consumers. Limited research has been performed to examine this issue. In one study [19], dietary intake of differently fed salmon (100% fish oil (FO), 50/50 FO/rapeseed oil, 100% rapeseed oil) and the influence on markers of human atherosclerosis were compared. Significant differences between the human consumer groups were observed in the serum fatty acid profiles, especially for the levels of total omega-3 PUFA and the omega-3/omega-6 ratio, which were markedly increased in the FO-fed fish consuming group in contrast to the two other groups. In addition, significant reductions of serum TAG and of vascular cell adhesion molecule-1 and interleukin-6 were observed in patients receiving the FO-fed salmon diet when compared with the two other groups. The authors concluded that Atlantic salmon fed the FO-containing diet containing very high concentrations of omega-3 LC-PUFA seemed to produce favourable biochemical changes in patients with coronary heart disease risk factors when compared with ingestion of fillets with intermediate and low levels of the marine omega-3 LC-PUFA, where FO was replaced in part or in full by rapeseed oil [19]. To our knowledge, there have been no consumer trials with fish fed diets containing LOA, ALA and/or SDA rich oils *versus* FO derived EPA + DHA, and looking at the effects on consumers.

A recent study tested whether Atlantic salmon smolt fed a diet with a higher DHA/EPA ratio and a lower content of LC omega-3 oils to that of conventional FO based diets would enhance deposition of LC omega-3 in the liver and muscle [20]. Comparisons were made between fish fed: (1) a FO diet; (2) a blend of 50% rapeseed and 50% tuna oil diet (termed model oil, MO1); (3) a blend of 50% rapeseed, 25% tuna and 25% FO diet (MO2); and (4) a blend of 50% FO and 50% poultry oil diet (FO/PO). The latter diet was representative of commercial diets in use in Australia at the time of the study, with the proportion of chicken fat increasing even further since the study was performed. The dietary DHA/EPA ratio was in the order MO1 > MO2 > FO/PO ~ FO. The LC omega-3 content was approximately 2-fold lower in the MO1, MO2 and FO/PO diets compared to the FO diet, with the relative levels (as % total FA) lowest in the MO1 diet. For the feeding trial, there were comparable contents of LC omega-3 in the muscle of the FO, MO 1 and FO/PO fed fish [20].

A major outcome for the feeding trial was the observation that a higher DHA/EPA ratio than that commonly occurring with FO-only diets used for Atlantic salmon was better suited for more efficient deposition of LC omega-3 in the flesh, in particular DHA. Evidence was therefore apparent for LC omega-3 “sparing” in the Atlantic salmon smolt fed a diet with a high DHA/EPA ratio [20]. The use of a 50% FO and 50% PO blend in aquafeeds for Atlantic salmon, as was in the range commercially practiced in Australia in 2010, resulted in comparable LC omega-3 content in the muscle [20] and liver of juvenile Atlantic salmon to a FO fed fish. It is noteworthy that such an oil blend decreases the inefficient utilization of a 100% FO diet, due to the high loss of EPA in particular, and may be considered as an appropriate current strategy, in terms of LC omega-3 sparing, for present use in aquafeeds for Atlantic salmon [20]. The reduction of FO incorporation in the aquafeeds has also enhanced the sustainability of the industry, although sufficient FO still remains in the feeds used to ensure that farmed Tasmanian Atlantic salmon remains one of the best sources of the LC omega-3 oils available to Australian consumers.

It is important to note that in spite of changes that have occurred in feeding practices, and the resulting lower content of LC omega-3 oils, the scope remains for the potential future use of new alternate sources of LC omega-3 to restore the content of these health-benefitting ingredients to those higher contents previously seen.

Further research is needed to determine the optimum relative and absolute concentrations of dietary EPA and DHA to enhance their deposition in larger-sized commercially farmed Atlantic salmon. The rationale to pursue such studies is supported by recent developments in plant genomics. As this research field has progressed, important breakthrough steps have included: the isolation and characterization of genes from the marine microalgae encoding front-end desaturases involved in DHA biosynthesis, the isolation of highly efficient desaturases and elongases, the use of genes with omega-3 substrate preference and the development and use of a land plant (tobacco) leaf-based assay using interchangeable design principles to rapidly assemble multistep recombinant pathways [21,22,23]. Progress with research on insertion of microalgal-derived genes leading to DHA production into a range of omega-3 C_18_ PUFA accumulating land plants has been reviewed [24,25,26].

Recent developments have resulted in oilseeds containing elevated LC omega-3 oils, including with fish oil-like proportions of DHA and an elevated omega-3/omega-6 ratio (2–5) [22,23,27]. These major breakthroughs can in the future provide the feed and aquaculture industries with an opportunity to both sustainably farm this key protein source for the growing global population, and enhance the nutritional quality of farmed seafood products in terms of the health-benefitting LC omega-3 oils. Alternate feed-grade oils containing the desired FA profiles, in particular including a high DHA/EPA ratio, are not presently available. However, a realistic examination of the future steps required suggests that they can be a commercial reality by the end of this decade [22,23].

### 4.2. Fish Oil Capsules as Sources of EPA + DHA

**Figure 4:**
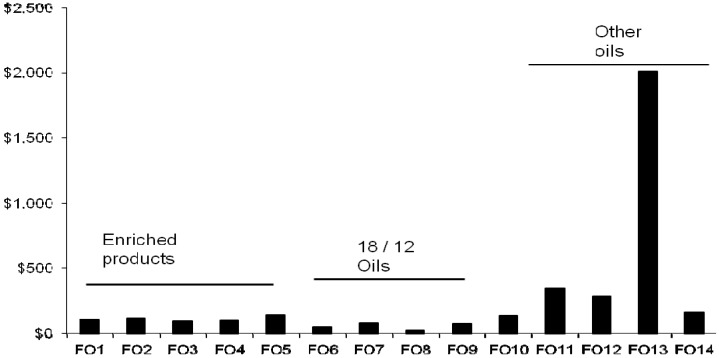
Cost per annum of fish oil products to supply 500 mg/day EPA + DHA. Sample codes refer to brands shown in Table 3. Enriched products—denotes group 1 brands containing concentrated EPA and/or DHA. 18/12 oils denotes—group 2 standard fish oils containing 180 mg/120 mg of EPA and DHA respectively. Other oils denotes—group 3 products containing varying proportions of EPA and DHA and may contain other components.

All products generally contained EPA + DHA at levels indicated on the product labels. The cost per 500 mg EPA + DHA (value generally advised for daily consumption by many nutritional and health groups [2]) varied markedly from $0.05 to $5.50 (Table 3), or from $20 to $2000 per annum (Figure 4). The lowest cost products, based on EPA + DHA levels alone, were those oils containing the conventional 180 mg EPA and 120 mg DHA per 1000 mg capsule (group 2). In the group 2 products, a four-fold cost difference occurred ($20–$79 pa, Table 3), although product specifications were identical. Consumers can have difficulties in compliance (e.g., consumption of multiple large capsules), and may prefer the range of enriched products (group 1) now available. The most expensive product based on EPA + DHA content was the krill oil, which was six-fold more expensive than the next most costly product.

## 5. Conclusions

In summary, in comparison to 2002 samples of two major farmed Australian finfish species, Atlantic salmon and barramundi sampled in 2010–2013 have been shown to contain decreased relative levels and content of LC omega-3 oils (from 2014 mg/100 g in 2002 decreasing to 975 mg/100 g in spring 2013 for Atlantic salmon; 1970 mg/100 g in 2002 decreasing to 790 mg/100 g per serve for barramundi). These changes have resulted from the use of new, lower cost and sustainable ingredients in farmed fish feed, necessitated by the inability of existing supplies together with the increasing cost of the wild harvest fish oil resource to meet the expanding needs of the aquaculture industry. Notwithstanding, these two widely available farmed fish species still remain an excellent source of the LC omega-3 oils, and in a broader context remain one of the best of all foods available for Australian consumers. All fish oil capsule supplements examined generally contained EPA + DHA at levels indicated on the product labels and, similar to the farmed seafood, such products can also represent a viable alternative source of LC omega-3 oils for consumers.

Subject to consumer demand, one of the cost-effective strategies to increase the omega-3 content of the lipid profile of farmed fish could be to include enhanced levels of omega-3 rich vegetable oils, and/or finishing diets with higher inclusions of marine oils [6]; such a strategy may result in grades of farmed seafood product being available for purchase that contain higher content of LC omega-3 than standard products grown using a predominately non-marine oil based diet. In the longer term, other strategies to consider include: selection for enhanced delta-5 and delta-6 desaturase activities (the key enzymes converting shorter chain omega-3 to EPA and DHA) and the use of alternate long-chain omega-3 oil sources, including new oil seeds containing EPA and DHA.
